# New Appliances and Things Medical

**Published:** 1903-11-14

**Authors:** 


### NEW APPLIANCES AND THINGS MEDICAL.

[We shall be glad to receive at our Office, 28 & 29 Southampton Street, Strand, London, W.C., from the manufacturers, specimens of all new preparations and appliances which may be brought out from time to time.]

### VALLS' BEETLECUTE.

(Valls and Co., 16 Coleman Street, London, E.C. Wholesale Agents: Burgoyne, Burbidges and Co., 12 and 16 Coleman Street, E.C.)

This preparation is a white, odourless powder intended to act as an exterminator of beetles, cockroaches, ants, woodlice, and other pests which infest kitchens, gardens, breweries, etc. After practical trials we are fully assured that its exterminating power is swift and certain, while its freedom from the dangerous ingredients contained in so many insect destroyers, rendering it harmless to human beings and domestic animals, is an important recommendation. "Beetlecute," should be welcome to all who need an efficient and reliable insecticide.

### DR. HUNTER'S IDEAL PEPSINIZED FOOD.

("Pharma" Food Company, Bermondsey Street, London. James Taylor, 132 Trongate Street, Glasgow.)

We must take exception to the statement that this food is an excellent substitute for mother's milk, for the inclusion of pepsin, though it is undoubtedly of use to infants in a certain number of selected cases and to those whose digestive functions are in an atonic condition, is not by any means desirable in the routine dietary of the very young. Our examination shows it to be somewhat malted and decidedly palatable, while its being already cooked renders it capable of being easily and rapidly prepared. In certain conditions of the digestive organs, therefore, this food should be of considerable use.

### WRIGHT'S COAL TAR SOAP, ETC.

(Wright, Layman and Umney, Limited, Southwark, London, S.E.)

This soap has been in popular use for many years, and our recent examination of it shows that it merits the position it holds in public favour. The proportion of water contained in it is small and the admixture of earthy matter reduced to a minimum, while the phenol-phthalein test shows an alcoholic solution to contain practically no free alkali. Though the importance of medicated soaps in dermato-therapeutics has been much exaggerated, and especially so in the case of antiseptic soaps, yet there is no doubt but that the preparation before us does exercise a healthy action on the skin and can be highly recommended for toilet use, and especially when irritating eruptions exist. A cruder and less refined soap is manufactured for scrubbing floors, etc., for which purposes its germicidal properties are no doubt salutary. A dentrifice and shaving soap are also prepared from the same constituents. Their mild antiseptic qualities and their pleasant odour make them most acceptable.

